# Implementing martial arts education in Chinese schools: teachers' perspectives on the school martial arts program

**DOI:** 10.3389/fspor.2025.1699131

**Published:** 2025-12-04

**Authors:** Shengwen Xue, Honglin Ji, Jikai Yang, Li Zhao, Xiangguo Su

**Affiliations:** 1 College of Physical Education, Shanghai Normal University, Shanghai, China; 2 Department of Physical Education, Qingdao Institute of Technology, Qingdao, China; 3 School of Sports Management and Communication, Capital University of Physical Education and Sports, Beijing, China

**Keywords:** school martial arts program, martial arts instruction, grounded theory, teacher participation, combat skills, cultivates character, teacher evaluation, implementation constraints

## Abstract

**Purpose:**

Martial arts activities can provide multiple benefits to adolescents. However, their educational effectiveness depends largely on teachers, who play a crucial role. From the perspective of Chinese teachers, this study explores teachers' choices and perceptions regarding the implementation of martial arts in primary and secondary schools.

**Method:**

Data were collected through semi-structured interviews with 25 Chinese primary and secondary school teachers who had exposure to the School Martial Arts Project (SMAP). The materials were analyzed using Strauss and Corbin's procedural grounded theory approach.

**Results:**

This study identified three themes: (a) Teacher evaluations: encompassing four aspects—difficulty, risks, effort-reward ratio, and value; (b) Constraints on teachers' implementation: including constraints from educational stakeholder groups and implementation conditions; (c) Teacher participation: manifesting in four participation modes: effective participation, implicit resistance, explicit opposition, and structural constraint.

**Conclusion:**

When confronted with the SMAP that emphasizes combat skills and nurturing character, they first assess the project's implementation difficulty, teaching risks, effort-reward ratio, and the extent to which educational value is realized. Teachers' participation is further influenced by several factors, including their martial arts competence, available facilities, the curriculum system, and the attitudes of educational stakeholders. The combined effects of these factors lead to variations in teachers' participation attitudes and behaviours, ultimately forming four participation modes: effective participation, implicit resistance, explicit opposition, and structural constraint. Teacher participation is a key determinant of SMAP's successful implementation, and comprehensive institutional support (e.g., training and facility assurances) is essential for promoting effective teacher engagement.

## 1 Introduction

Martial arts have been associated with a range of positive developmental outcomes among adolescents (1). Research consistently shows that martial arts training enhances physical competence—such as cardiovascular fitness and motor skill development—while also fostering psychological and socio-emotional growth, including gains in self-efficacy (2), emotional regulation (3), cooperative behaviour, and reductions in aggression (4, 5). Moreover, martial arts have been linked to improvements in cognitive functions, particularly executive functioning (6, 7), as well as modest gains in academic achievement (8). Recent systematic reviews further underscore the psychological and developmental benefits of martial arts for children and adolescents with disabilities, reporting improvements in mental health, executive functioning, and social inclusion (9, 10). Collectively, these findings support the feasibility and acceptability of integrating martial arts into school physical education curricula and broader educational settings (11). Nevertheless, some studies raise concerns about safety risks associated with martial arts, particularly the potential for such activities to increase aggressive behaviours among students. These concerns constitute a significant barrier to the wider adoption of martial arts programmes in school settings (12, 13).

Historically, martial arts were not developed as educational tools for children or formal school settings (14). Nevertheless, various martial arts—such as tai chi, taekwondo, fencing, and capoeira—have been successfully adapted for use in school contexts (15–18). To support the systematic integration of martial arts into physical education, scholars have proposed pedagogical frameworks focusing on content organization and instructional methods. For example, Espartero proposed categorizing martial arts into three types: grappling, striking, and weapon-based forms (19). Gomes and Rufino (20, 21) further classified martial arts according to interaction distance: short-distance (e.g., wrestling), medium-distance (e.g., boxing and kicking), long-distance (e.g., fencing), and mixed-distance (e.g., mixed martial arts). In the domain of pedagogy, Del Vecchio (22) proposed adapting Claude Bayer's “pendulum model” for introducing martial arts to children aged seven and above. This model outlines a three-stage progression: (1) play-based combat games for ages seven–ten; (2) emphasis on rules and movement patterns for ages 10–12; and (3) instruction in specific martial arts techniques for adolescents aged 13 and older. Breed and Rufino (19, 23) proposed a gamified instructional approach to martial arts teaching, integrating tactical models with principles of gamification.

Researchers have proposed various effective strategies for the successful implementation of martial arts in school settings, while also recognizing the critical role of teachers in facilitating martial arts education. Vertonghen found that adolescent participation in martial arts can yield contradictory outcomes, ranging from highly positive to highly negative. These effects are influenced by underlying conditions, with the teacher's preferred martial arts teaching approach being a core contextual factor (24). Kooi discovered that the balance of “strictness and empathy” in martial arts instructors is essential for fostering adolescent development: strictness helps adolescents build discipline and perseverance, while empathy enhances students' trust and sense of belonging, encouraging active engagement. This balance distinguishes martial arts instructors from other physical education teachers (25). Theeboom found that merely providing martial arts programmes for socially disadvantaged youth is insufficient to ensure positive outcomes; the teacher's fundamental qualities and instructional processes are critical (26).

A large body of literature has currently explored the positive effects of martial arts on promoting adolescent development. Given that teachers act as intermediaries in translating the educational potential of martial arts into classroom practice, understanding their perceptions and engagement is crucial for ensuring the effective and safe implementation of martial arts programmes. However, few studies have focused on teachers' perspectives; therefore, research on teachers' attitudes and perceptions regarding the implementation of this sport program in primary and secondary schools should be conducted. In China, the National School Martial Arts Alliance (NSMA) was established in 2013 to promote martial arts education in schools. It is administered by the Department of Physical Education, Health, and Art Education at the Ministry of Education and led by Shanghai University of Sport. The alliance introduced a reform philosophy for school-based martial arts, encompassing four core principles: “One School, One Martial Arts Style” (each school selects and promotes a distinct martial arts style according to its characteristics); “Integrating practice with instruction” (emphasizing the integration of foundational training with practical combat skills); “Integrating technique and philosophy” (integrating martial arts instruction with the values of traditional Chinese culture); and “Cultivating both virtue and skill” (enhancing students' technical proficiency while nurturing their moral character). This initiative is also known as the School Martial Arts Program (SMAP), guided by the teaching philosophy of “emphasizing combat skills and nurturing character”. Since the establishment of the NSMA in 2013, SMAP has evolved into a national curriculum reform initiative. Given its expanding implementation, there is an urgent need for empirical research on teachers' participation and their role in delivering the program effectively. The main research questions are: How do teachers view the integration of martial arts into school physical education curricula? What behaviours do they adopt in response to school martial arts programmes? The findings of this study can provide new insights for the promotion and improvement of martial arts in schools.

## 2 Materials and methods

### 2.1 Methods

This study employed a grounded theory approach, which is well-suited for constructing theories to explain specific processes or to abstractly generalize human behaviours (27). Among the main variants of grounded theory, we adopted Strauss and Corbin's procedural grounded theory (28, 29), which emphasizes change, conditions, processes, and actions, and elucidates phenomena through relational statements. Consequently, our paradigmatic stance shares significant common ground with the critical realism perspective advocated by Bhaskar, Maxwell, and Sayer (30–33). A key feature of critical realism is the integration of realist ontology with interpretive epistemology. This compatibility stems from critical realism's realist ontology—positing an objective reality shaped by underlying mechanisms and structures—aligning with procedural grounded theory's interpretive epistemology, which emphasises emergent understanding from data. This alignment supports the study's analytical goal of uncovering relational dynamics in teachers' participation in SMAP through the interplay of events, conditions, and outcomes. It is important to note the influence of our critical realist perspective on this study. In critical realism, explanations of events and their causal relationships do not rely on the regular succession or repetition of events, as in positivism, but rather on the interplay of mechanisms, structures, and conditions (33). Thus, critical realism provides a philosophical foundation for this grounded theory study, enabling theory construction through the conceptualization of events, mechanisms, structures, and their interrelationships.

### 2.2 Context and sample selection

This study recruited teachers from China, where efforts have been made to promote martial arts in primary and secondary schools. In 2013, under the leadership of the Department of Physical Education, Health, and Arts Education of the Ministry of Education, and with Shanghai University of Sport as the leading institution, the National School Sports Alliance (Chinese Martial Arts), also known as the National School Martial Arts Alliance, was established. The alliance proposed a reform concept for school martial arts encapsulated in the principle of “one school, one style; integrating practice with instruction; integrating technique and philosophy; cultivating both virtue and skill”. This concept emphasizes incorporating the combative (fighting) elements of traditional Chinese martial arts into teaching content to foster students' character development, referred to as the SMAP, which prioritizes “emphasizing combat skills to nurture character”. The alliance actively supports its member institutions across various provinces and cities in implementing SMAP, providing a rich source of participants for this study. The first author and corresponding author, affiliated with a member institution of the alliance, were well-positioned to conveniently and comprehensively collect teacher samples.

Teachers were invited to participate in this study via emails sent through the secretariat of the National School Martial Arts Alliance. In the first year, we selected five participants from a list of teachers who expressed interest in the email, based on the extent of their physical education teaching experience and the geographical distribution of their affiliated member institutions. This initial selection served two purposes: first, to conduct preliminary interviews to prepare for formal interviews, with data from these preliminary interviews excluded from the formal analysis; and second, to gain an in-depth understanding of their specific practices in SMAP and establish close collaboration with them. This consideration was critical, as the first year of the study required laying a solid foundation for subsequent work.

Following the principles of theoretical sampling in grounded theory, purposive sampling was employed instead of random sampling to select participants who could provide novel attributes or dimensions (e.g., “teachers with failed SMAP implementation experiences” or “teachers instructing both martial arts and other physical education subjects”). Participants were primarily recruited from regions with concentrated membership in the National School Martial Arts Alliance: Shandong, Zhejiang, Jiangsu provinces, and Shanghai municipality. Initially, 32 teachers were invited to participate, but seven declined (a 21.9% refusal rate), with no attrition after enrollment. Reasons for refusal included heavy teaching workloads limiting available time or concerns about disclosing sensitive pedagogical practices. Ultimately, 25 teachers constituted the final sample. Table 1 details participants' teaching experience duration, educational stages taught, and associated martial arts schools. Additional demographic characteristics include: 14 males (56%) and 11 females (44%); teaching levels spanning elementary schools (*n* = nine, 36%), junior high schools (*n* = eight, 32%), and senior high schools (*n* = eight, 32%).

**Table 1:** Basic information of interviewees.

Interviewee	Experience	School location	Purpose	Martial arts style
A1	10	Jiangsu	Theoretical formulation	Xingyi Quan
A2	5	Jiangsu	Theoretical formulation	Taijiquan
A3	6	Jiangsu	Theoretical formulation	Taijiquan
A4	10	Jiangsu	Theoretical formulation	Bajiquan
A5	3	Shandong	Theoretical formulation	Bajiquan
A6	6	Shanghai	Theoretical formulation	Taijiquan
A7	7	Zhejiang	Theoretical formulation	Xingyi Quan
A8	8	Zhejiang	Theoretical formulation	Wushu Sanda
A9	8	Zhejiang	Theoretical formulation	Taijiquan
A10	11	Shandong	Theoretical formulation	Xingyi Quan
A11	10	Jiangsu	Theoretical formulation	Bajiquan
A12	3	Shandong	Theoretical formulation	Taijiquan
A13	3	Shandong	Theoretical formulation	Tanglangquan
A14	5	Shanghai	Theoretical formulation	Wushu Sanda
A15	17	Shanghai	Theoretical formulation	Taijiquan
A16	9	Jiangsu	Theoretical formulation	Taijiquan
A17	3	Shandong	Theoretical formulation	Sanpu Longquan
A18	3	Shandong	Theoretical formulation	Taijiquan
A19	6	Shanghai	Theoretical formulation	Wushu Sanda
A20	8	Shanghai	Theoretical formulation	Bajiquan
A21	5	Jiangsu	Theoretical formulation	Xingyi Quan
A22	6	Shandong	Validation stage	Wushu Sanda
A23	4	Shandong	Validation stage	Sanpu Longquan
A24	5	Shanghai	Validation stage	Wushu Sanda
A25	11	Shanghai	Validation stage	Taijiquan

### 2.3 Data collection

Data were primarily collected through face-to-face or audio interviews guided by a semi-structured interview protocol. The interviews were conducted collaboratively by the first author and the corresponding author. The first author, a martial arts specialist with a master's degree and seven years of research experience in school physical education, had participated in three school martial arts education projects. The corresponding author, a professor in martial arts education with 20 years of research experience, had led two provincial-level school martial arts reform initiatives. Data collection settings were as follows: Face-to-face interviews were conducted in private meeting rooms or physical education offices at participants' workplaces to protect participant privacy, avoiding public areas or instructional settings. Audio interviews were administered via Tencent Meeting platform with pre-tested network stability to ensure data qualityThe interview protocol consisted of three core questions:

a. How do you view the implementation of martial arts programmes in schools?
b. If you were to implement a school martial arts program, what actions would you take?
c. What factors would you consider when implementing martial arts teaching in schools, and what influences might affect this process?

The interview guide underwent pilot testing through trial interviews with five teachers not included in the final sample. Based on participant feedback, question phrasing was revised to enhance neutrality and clarity. For instance, the original question “Do you support SMAP?” was reformulated as “How do you perceive the implementation of SMAP?” to avoid leading language. Ambiguous terminology was systematically clarified through iterative refinements, ensuring both neutrality in question framing and readability for diverse educational contexts.

While the core questions of the interview guide remained consistent, researchers employed dynamic probing based on emerging concepts and categories derived from preliminary data analysis, as well as participants' responses. All audio recordings were transcribed verbatim and subjected to a participant validation process: de-identified transcripts (with anonymized identifiers including school names, teacher identities, and sensitive institutional details) were returned to corresponding participants within one week post-interview. Respondents were granted one week to review materials and propose modifications, though none requested textual revisions upon final verification. The study yielded 25 audio recordings with an average interview duration of 30.6 min, generating transcribed texts averaging 4,000 words per case (totaling 102,500 words). Qualitative data analysis was conducted using NVivo 12.0 Plus, with iterative coding procedures informing theoretical saturation and guiding subsequent sampling strategies.

### 2.4 Trustworthiness

The study employed a process of group-based analysis, validation with additional interview samples, and subsequent verification. First, the research team, consisting of four members, was divided into two groups to independently analyze the data. After completing their analyses, the groups conducted a unified comparison and made necessary adjustments. Second, following the initial theory generation, an additional sample of four teachers was collected to test the theory. The concepts and categories derived from this additional sample were effectively integrated into the previously constructed theory. Subsequently, the generated theory and related materials were shared with the interviewed teachers for feedback, and they expressed agreement with the proposed theory. This process ensured that the data did not fall into a state of “false saturation” (34).

### 2.5 Reflexive statement of researchers

In accordance with the requirements of reflexivity in qualitative research (35, 36), this study must clarify the researchers' positionality, potential assumptions, and their impacts on the research process to enhance transparency and the credibility of results:

Researchers'Background and Positionality:The first author and corresponding author are both affiliated with member institutions of the National School Martial Arts Alliance, with long-term engagement in research on school physical education and martial arts education, as well as participation in the local promotion of SMAP. This background offers advantages: it enables quick comprehension of the teaching context in teacher interviews (e.g., “difficulties in combat skills teaching”, “conflicts between administrative requirements and teaching practice”) and reduces communication barriers. However, there is a potential risk of a preliminary assumption of “tendency to approve of SMAP's value”. To balance this tendency, the research team conducted pre-interview calibration before formal interviews—conducting pilot interviews with 5 non-sample teachers to verify the neutrality of the interview outline—and adopted cross-validation during the data analysis phase.

Composition of the Research Team and Bias Control:The research team consists of six members, including two physical education researchers (focusing on theoretical analysis), two frontline coordinators of SMAP (focusing on interpretation of practical contexts), and two experts in qualitative research methods (focusing on control of analytical logic). During the coding stage, the team was divided into two groups for independent analysis; differences were then resolved through group discussions to avoid biases caused by a single perspective. Reflections on Data Collection and Analysis:During interviews, researchers avoided leading questions such as “Do you support SMAP?” and instead used open-ended questions like “What is your view on the implementation of SMAP?” When transcribing texts, “negative feedback mentioned by teachers” (e.g., “SMAP increases teaching burden”) was fully retained and not omitted for being “inconsistent with presuppositions”. During the theoretical saturation test stage, teachers who “hold negative attitudes toward SMAP” were proactively selected as supplementary samples to ensure the theory covers different perspectives.

### 2.6 Ethical considerations and procedural details

This study strictly adhered to the Ethical Review Procedures for Biomedical Research Involving Human Subjects. All procedures received ethical approval from the Academic Ethics and Morality Committee of Shanghai Normal University (Approval No.: 2025108; Approval Date: August 15, 2023; Approval Location: No. 500 Qinzhou South Road, Xuhui District, Shanghai).Specific ethical protocols included:Informed Consent:A written Informed Consent Form was provided to each participant prior to interviews, detailing the study's purpose, content, data usage (strictly for academic research with anonymized processing), and participation rights (voluntary withdrawal without negative consequences). Interviews commenced only after voluntary signature by teachers. Privacy Protection:Interview audio recordings were securely stored by core research team members only. Transcribed texts removed identifiable information (e.g., school names, teacher identities, contact details) and used anonymized codes (“A1, A2”) for participant identification. Result Feedback:When sharing findings with participants, individual perspectives were aggregated to ensure non-disclosure of other teachers' perceptions, maintaining strict confidentiality of personal data.

## 3 Results

### 3.1 Open coding

Open coding yielded eight main categories (see Table 2). Based on thematic relevance, these eight categories coalesce into three overarching dimensions. The first is the Teacher Evaluation dimension—encompassing difficulty evaluation, risk evaluation, value evaluation, and cost-benefit evaluation—which reflects teachers’ initial assessments of the feasibility of SMAP implementation. The second is the Teacher Implementation Constraints dimension, comprising constraints related to educational stakeholders and implementation conditions. This dimension captures the external and internal barriers that influence teachers' participation in SMAP. The third is the Teacher Participation dimension, consisting of attitudinal support and behavioural participation. This dimension represents the outcome of the interplay between the first two dimensions—teachers’ evaluations and perceived constraints—in shaping their engagement with SMAP.

**Table 2:** Conceptualization and categorization of open coding.

Category	Subcategory	Initial concept
C1 Difficulty evaluation	B1 Preparation Difficulty Estimation	Aa1 Difficulty in designing teaching plans, Aa2 Difficulty in conducting martial arts teaching
	B2 Consideration of Teaching Difficulty	Aa3 Difficulty in Considering Safe Teaching, Aa4Difficulty in Considering Effective Teaching
C2 Risk Evaluation	B3 Prediction of Teaching Risks	Aa5 Teaching risk of competitive practice, Aa6 Classroom management risk
	B4 Avoidance of Teaching Risks	Aa7 Reducing teaching risks through hardware Aa8Rational Org. for Teaching Risk Red.
C3 Value Evaluation	B5 Evaluation of Positive Value	Aa9: Character Cultivation; Aa10: Self-Defense; Aa11: National Cultural Identity
	B6 Evaluation of Negative Value	Aa12: Student Injuries; Aa13: Aggressive Behavior; Aa14: Campus Violence Incidents
C4 Cost-Benefit Prediction	B7 Input Prediction	Aa15 Adaptation to martial arts teaching concepts, Aa16 Participation in training and teaching research, Aa17 Reconstruction of teaching content
	B8 Benefit Prediction	Aa18 Performance benefits, Aa19 Professional title promotion benefits, Aa20 Reputational benefits
C5 Constraints from Educational Stakeholders	B9 Student Responses	Aa21 Feedback on martial arts routine learning, Aa22 Expectation for martial arts combat skills
	B10 Parents’ Perspectives	Aa23 Parents’ cognition of martial arts safety, Aa24 Parents’ cognition of martial arts value
	B11 School Leaders’ Requirements	Aa25 Administrators’ decision - making power, Aa26 Safety requirements for martial arts combat skills, Aa27 Administrators’ cognition of martial arts combat skills value
C6 Constraints on Implementation Conditions	B12 Limitations on Combat Skills	Aa28: Insufficient Martial arts Combat Skills; Aa29: Insufficient Martial arts Teaching Skills
	B13 Limitations on martial arts Teaching System	Aa30: Lack of Teaching Methods; Aa31: Lack of Knowledge on Teaching Objectives; Aa32: Lack of Knowledge on Teaching Content; Aa33: Lack of Knowledge on Teaching Evaluation
	B14 Limitations on Hardware Facilities	Aa34 Venue limitations, Aa35 Equipment limitations
	B15 Lack of In - service Training	Aa36 Lack of training on martial arts teaching ability, Aa37 Lack of special training on martial arts teaching skills
	B16 Lack of Teaching Seminars	Aa38: Lack of Study on Martial arts Teaching Methods; Aa39: Lack of Study on Martial arts Teaching
	B17 Lack of Competition System	Aa40 Lack of martial arts combat competition system
C7 Attitudinal Support	B18 Attitudinal Support	Aa41 Psychological recognition, Aa42 Emotional support
	B19 Attitudinal Non-Support	Aa43: Psychological Disidentification; Aa44: Emotional Non-Support
C8 behavioural Participation	B20 Behavioural Participation	Aa45: Teaching behavioural Participation; Aa46: Non-Teaching behavioural Participation
	B21 Behavioural Non-Participation	Aa47: Teaching behavioural Non-Participation; Aa48: Non-Teaching behavioural Non-Participation

### 3.2 Axial coding

**Figure 1:**
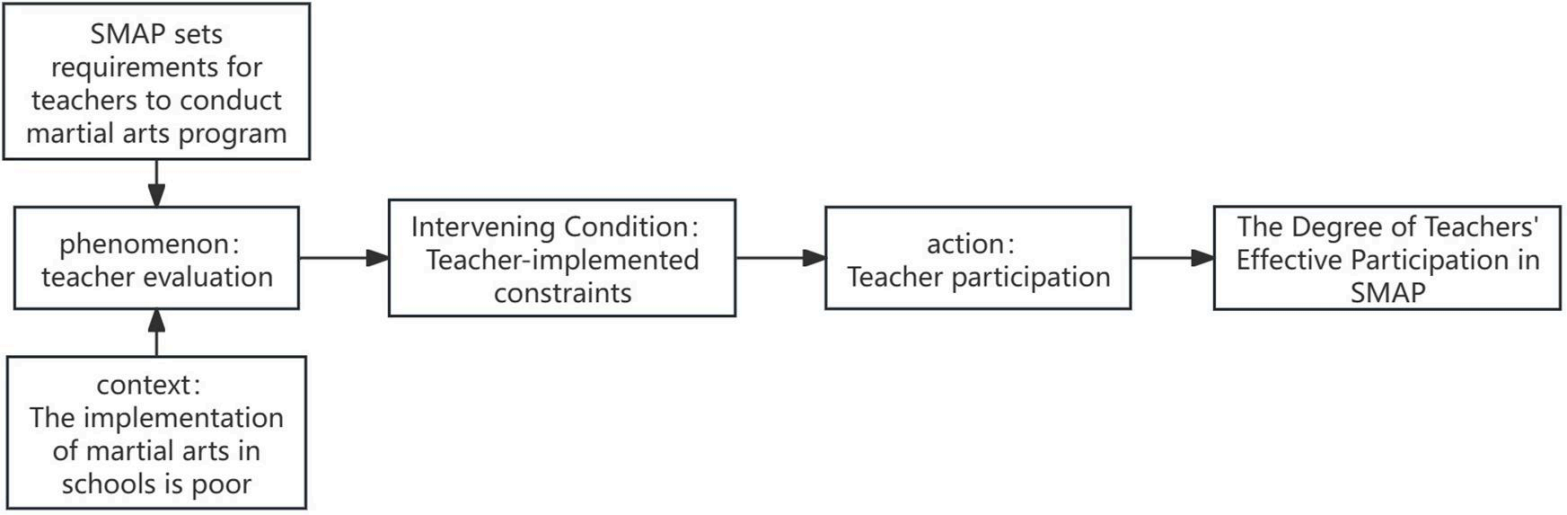
Paradigm model of teacher participation in the implementation of SMAP. Causal conditions correspond to teachers multi-dimensional evaluation of SMAP, Central Phenomenon refers to the participation decision-making dilemma faced by teachers, context is the conceptual requirement of SMAP, i.e., cultivating character through combat skills, intervening conditions are various constraints during teachers implementation process, actions are teachers’ specific participation behaviours, and outcomes are differentiated participation modes. This model clearly organizes the connection paths of each core category, providing structural support for the transition of grounded theory from “category division” to “systematic integration” and making the subsequent construction of the theoretical framework more logical.

Building on the eight initial categories identified through open coding, axial coding was employed to examine their interrelationships and integrate the fragmented concepts into a more coherent analytical framework. Specifically, difficulty evaluation, risk evaluation, value evaluation, and cost-benefit evaluation were grouped under the Teacher Evaluation dimension. Constraints related to educational stakeholders and implementation conditions were subsumed within the Teacher Implementation Constraints dimension. Attitudinal support and behavioural participation were integrated into the Teacher Participation dimension. These groupings are summarized in Table 3. Guided by Strauss and Corbin's paradigm model—structured around the core elements of causal conditions, context, intervening conditions, central phenomenon, actions, and outcomes—a relational analysis was conducted to identify linkages among the three dimensions and integrate the preliminary coding framework into a coherent theoretical system. The resulting theoretical model is illustrated in Figure 1.

**Table 3:** Main axis coding table.

Axial coding category	Open coding category	Category connotation
D1 Teacher Evaluation	C1 Difficulty Evaluation	Teachers assess the overall difficulty of implementing martial arts teaching concepts.
	C2 Risk Evaluation	Teachers assess the teaching risks of carrying out martial arts teaching.
	C3 Value Evaluation	Teachers assess how confident they are in achieving the martial arts education goals of “inheriting culture and cultivating morality” through martial arts teaching.
	C4 Cost - Benefit Evaluation	When teachers participate in SMAP, they measure the costs they need to pay and the possible benefits they can get.
D2 Constraints on Teacher Implementation	C5 Constraints from Education - related Systems	The perceptions of school leaders, parents, and students on teachers’ participation in SMAP that “emphasize combat and cultivate character” restrict teachers’ participation behavior.
	C6 Constraints from Implementation Conditions	The necessary conditions such as teaching ability, teaching system, hardware, and in - service training required to implement the concept of SMAP restrict teachers’ participation behavior.
D3 Teacher Participation	C7 Attitudinal Support	The attitude held by teachers towards the SMAP they participate in.
	C8 Behavioural Participation	The actual behavior of teachers participating in school martial arts teaching projects.

### 3.3 Selective coding

The core category was identified through the construction of an emergent narrative that integrated key participant experiences and conceptual linkages across the data. The SMAP, guided by the principle of “emphasizing combat skills and nurturing character”, requires teachers to design safe instructional plans and employ pedagogical approaches that integrate character development into martial arts instruction. The introduction of this new program has disrupted teachers' established physical education teaching practices. Shaped by the interplay of teachers' evaluative judgments, contextual implementation conditions, and pressures from educational stakeholders, teachers have developed diverse attitudes and behavioural responses toward SMAP. Based on the criteria of centrality, frequency, and explanatory power, Teacher Participation was established as the core category. Centrality: Teacher Participation emerges as the outcome of Teacher Evaluation and is shaped by Teacher Implementation Constraints. It functions as the central nexus linking these dimensions and is pivotal to explaining the mechanisms through which teachers engage with or resist SMAP implementation. Frequency: References to Teacher Participation—including expressions such as “willingness to participate”, “participatory behaviour”, and “concerns about participation”—recurred consistently across all 25 interview transcripts, appearing with greater frequency than any other category. Explanatory Power: Teacher Participation integrates the dynamic interplay between Evaluation and Constraints, enables the classification of distinct participation patterns (e.g., active engagement, reluctant compliance, or non-adoption), and comprehensively accounts for the range of teacher behaviours observed during SMAP implementation.

**Figure 2:**
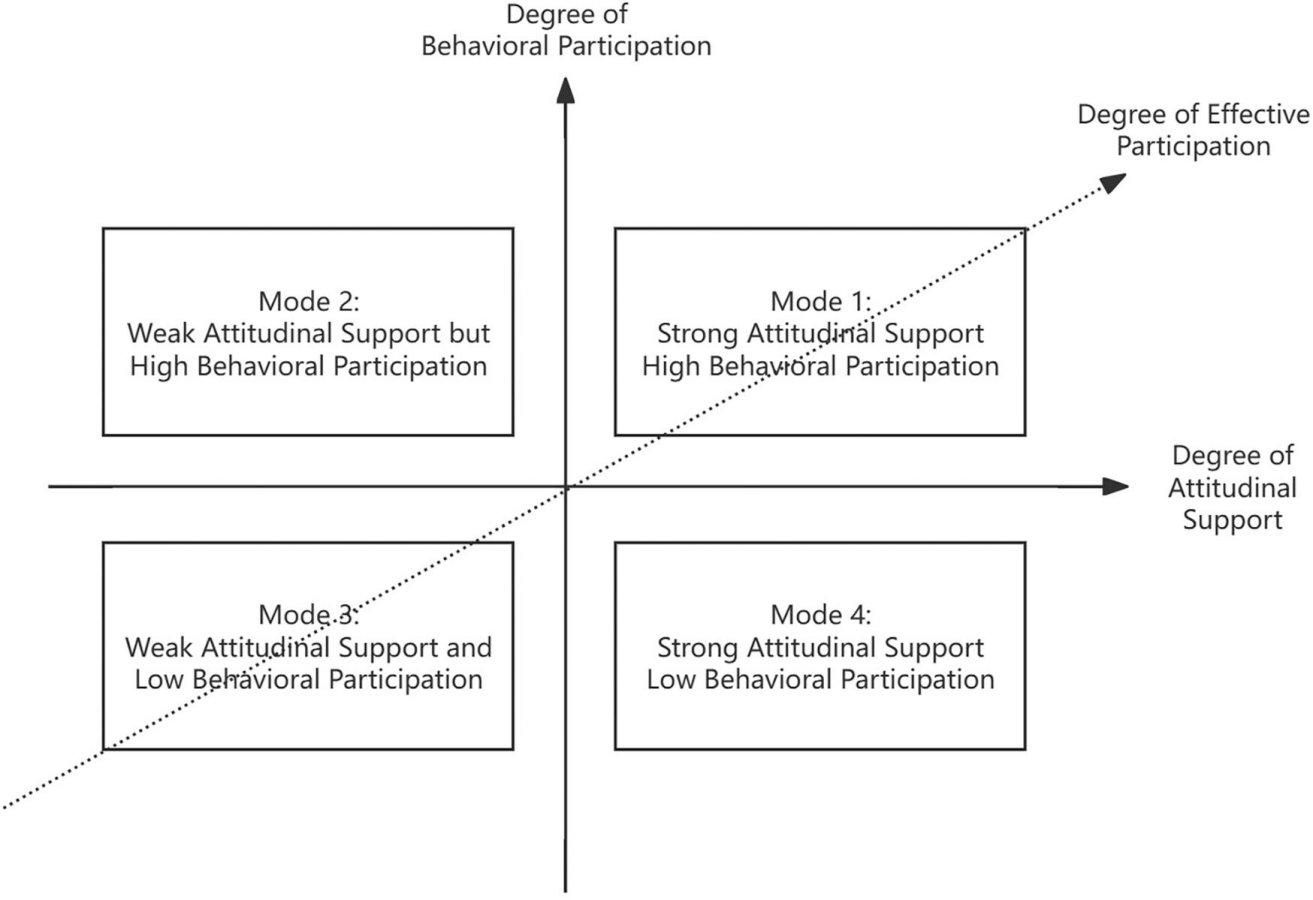
Core category analysis of teachers’ participation in SMAP. This figure classifies teachers’ participation into four modes via the intersection of Degree of Attitudinal Support (horizontal axis) and Degree of behavioural Participation (vertical axis). It visually illustrates the interactive relationship between teachers’ attitudes and behaviours under the core category of “teacher participation”, translating prior categories (such as teacher evaluation and implementation constraints) into differentiated manifestations of attitudes and behaviours. In doing so, it provides an intuitive framework for subsequently refining the causal mechanism of “evaluation-constraints → attitudes-behaviours’ in grounded theory, advancing the theory from category correlation toward mechanism interpretation.

Based on the varying degrees of teachers’ attitude support and behavioural engagement, different types of teacher participation emerged: (1) When both attitude support and behavioural engagement are high, teachers exhibit the highest level of assessment of the program's concept and face the lowest level of external constraints, resulting in the highest degree of effective participation and the greatest likelihood of implementing the “emphasizing combat skills to nurture character” concept. (2) When behavioural engagement is high but attitude support is low, teachers’ assessment of the program is low, indicating a lack of internal endorsement of the martial arts teaching concept; however, external pressures—particularly administrative mandates from school leadership—compel them to participate. (3) When both attitude support and behavioural engagement are low, teachers have a low assessment level and face significant external constraints, leading them to resist participation in SMAP both attitudinally and behaviourally. (4) When attitude support is high but behavioural engagement is low, teachers have a relatively high assessment level and endorse SMAP, but external conditions and pressures from stakeholder groups prevent them from implementing the corresponding teaching activities, resulting in an inability to participate in the reform. See Figure 2.

### 3.4 Theoretical framework for teacher participation in the school martial arts program

**Figure 3:**
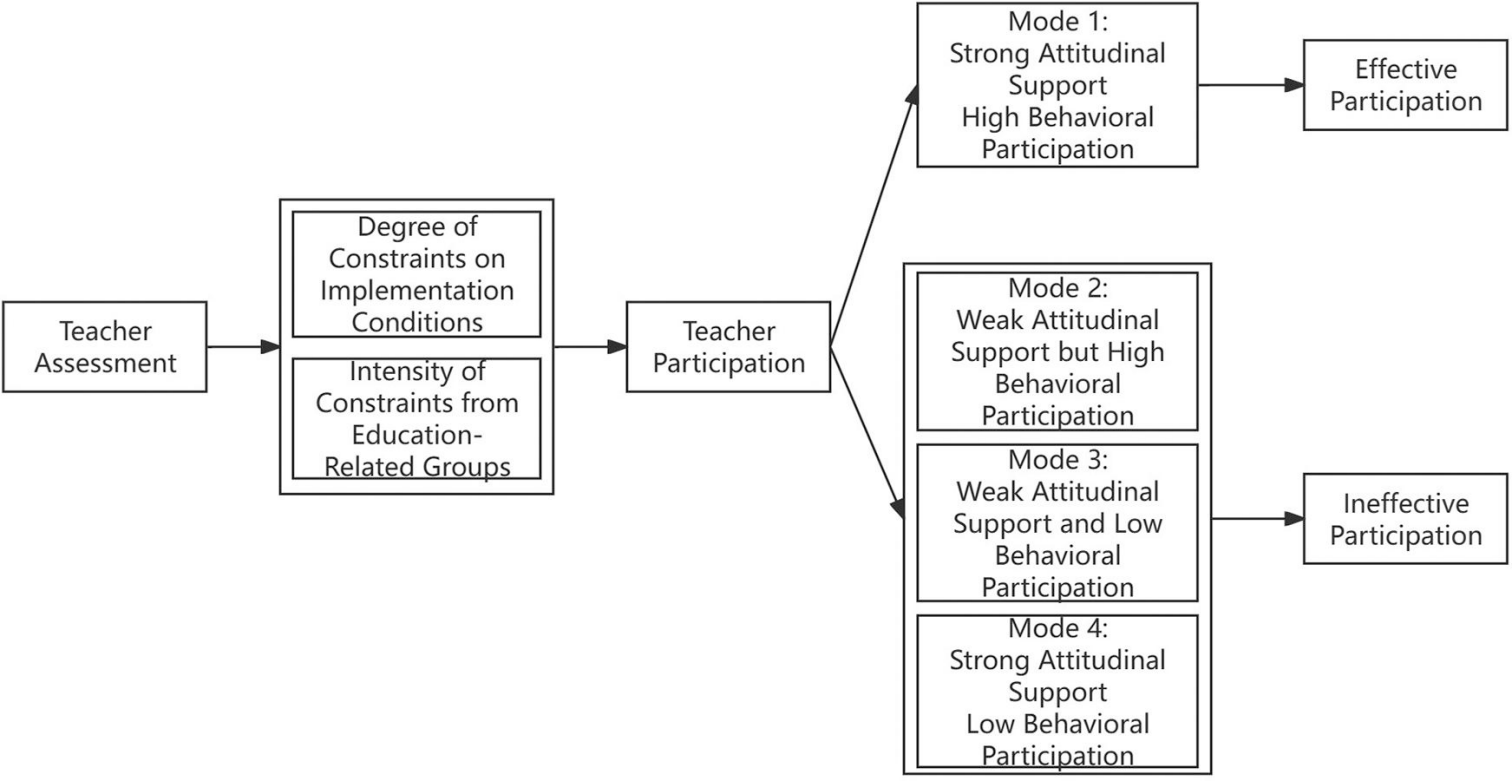
Teachers’ participation model in SMAP. Figure 3 presents the generation logic of teachers’ participation models in SMAP: Starting from “Teacher Evaluation”, it forms “Teacher Participation” through the dual effects of “Degree of Constraints on Implementation Conditions” and “Intensity of Constraints from Education-Related Groups”, then refines into four modes, and finally categorizes them into two types: “Effective Participation” and “Ineffective Participation”. This logic concretizes the “evaluation-constraints-participation” relationship in grounded theory into mode classification and result induction, providing a clear framework for theoretical saturation verification and promoting the theory from the stage of category integration to result verification.

The theoretical logic of teachers' participation in SMAP is as follows: Teacher Evaluation and implementation constraints jointly shape teachers' engagement, giving rise to four distinct participation patterns. effective participation: Teachers have a positive evaluation of SMAP, including low implementation difficulty, low teaching risk, high educational value and high cost-benefit ratio, and face weak Implementation Constraints, such as sufficient martial arts teaching ability, complete hardware facilities and support from students, parents and administrators; implicit resistance: Teachers have a negative evaluation of SMAP, including high safety risks and low value recognition, but are forced to participate due to strong implementation constraints, such as administrative requirements from school administrators; explicit opposition: Teachers have a negative evaluation of SMAP, including high time and energy input and low benefits, and face strong implementation constraints, such as no specialized training and insufficient teaching ability, thus actively refusing to participate;structural constraint: Teachers have a positive evaluation of SMAP, including recognition of the value of “nurturing character through combat skills” and low risk perception, but face strong implementation constraints, such as lack of protective gear or venues and no access to ability training, thus being unable to participate. Patterns 2, 3, and 4 hinder SMAP implementation and contribute to teacher resistance, making teacher participation the key determinant of resistance generation. Combined with insights from memo writing and selective coding, the following theoretical model was developed, as shown in Figure 3.

## 4 Analysis and discussion

### 4.1 Analysis of the core category “teacher participation”

As the core category, teacher participation serves as the ultimate expression of teachers' attitudes toward the integration of martial arts into school physical education curricula and their behaviours regarding the School Martial Arts Program (SMAP). When confronted with SMAP, which is guided by the concept of “emphasizing combat skills and nurturing character”, teachers do not accept or reject it outright; instead, they form their final participation behaviours through the dynamic interaction between Teacher Evaluation and Implementation Constraints. This process not only reflects teachers' rational decision-making but also embodies the interaction between individuals and their environment in educational reform—consistent with Ajzen's Theory of Planned Behavior, which posits that behaviours not entirely under personal control are influenced by both individual attitudes and perceived behavioural control arising from practical conditions (37). From the perspective of Priestley et al.'s ecological model of teacher agency, teachers are not passive recipients of reform; rather, they are active agents who construct their participation pathways through the interplay between structure (e.g., SMAP policies, hardware facilities, administrative mandates) and agency (e.g., evaluative judgments, responses to constraints) (38). Fullan's theory of educational reform implementation further emphasizes that curriculum reform is a complex, adaptive process shaped by the interplay of reform demands, teachers' professional alignment, and environmental support (39). The dynamic adjustment of teachers' participation behaviours in SMAP reflects this complexity, accounting for the observed variations in how teachers engage with the program. These results align with prior literature on teacher agency and educational reform.

Building on the theoretical foundations of teacher participation in SMAP outlined above, the next section analyses the limitations of existing studies in this field. Existing studies have limitations in two aspects. First, they tend to focus on single influencing factors. For example, the lack of martial arts training in Physical Education Teacher Education (PETE) programmes leaves teachers without the ability to teach martial arts (40, 41); Tabet found that popular culture tends to make teachers worry about the association between martial arts and violence (42). However, these studies fail to attend to the comprehensive judgment made by teachers as active agents. Second, most studies present influencing factors from a cross-sectional perspective—for instance, the psychological concerns identified by Van (43) and the time and funding constraints highlighted by Brownell (44) —without dynamically analyzing the participation mechanism and process from teachers' own perspectives. Based on grounded theory, this study reveals that teachers' four-dimensional evaluation framework of “difficulty-risk-value-cost-benefit” breaks through the focus on static factors in the Theory of Planned Behavior and aligns with the dynamic logic of “iteration-projection-practical evaluation” in Priestley et al.'s theory of teacher agency (38). For instance, when facing SMAP, some teachers evaluate its feasibility based on “past martial arts teaching experience” (iteration dimension), plan programmes by considering “student safety and hardware conditions” (practical evaluation dimension), and move closer to the goal of “emphasizing combat skills and nurturing character” (projection dimension) by simplifying confrontational movements—this reflects the adaptive adjustment of agency under structural constraint. This also echoes Lasky's theory of teacher agency, which points out that teachers respond to policies through “interpretation-negotiation-reconstruction”. For example, “implicit resistance” is essentially a combination of strategies including “interpreting high risks in confrontational teaching, negotiating superficial compliance, and reconstructing by abandoning confrontational sessions”, highlighting the mediating role of teacher agency (45).

By examining the interplay between the three core themes, the findings clarify that teacher participation is the joint outcome of “evaluation laying the foundation for attitudes and constraints defining the boundaries of behaviours”:When the evaluation leans toward “high value + low constraints” (e.g., perceiving SMAP as easy to teach and safe) and the constraints are weak (e.g., schools providing training), “effective participation” is likely to occur; When the evaluation emphasizes “high risk + low benefit” (e.g., teachers worrying about “the risk of injuries due to lack of venues”) and the constraints are strong (e.g., no hardware support), “implicit resistance”, “explicit opposition”, or “structural constraint” tend to emerge. This finding not only echoes all dimensions of the Theory of Planned Behavior but also explains the variation in participation behaviours under similar constraint conditions within the framework of teacher agency: teachers” agentic interpretation of and response to constraints directly influence the nature and outcome of their participation.

With the criterion of whether the concept of “emphasizing combat skills and nurturing character” can be implemented, Teacher Participation Modes 2, 3, and 4 are identified as forms of teacher resistance in SMAP, with details as follows. Mode 2: implicit resistance (High behavioural Participation, Weak Attitudinal Support)Due to concerns about student injuries, teachers select martial arts routines as instructional content and adopt the “movement demonstration and imitation” method—similar to the “educational sporting teaching approach” proposed by Theeboom and Vertonghen (41, 46). However, they omit sessions related to practical application and offensive-defensive confrontation (e.g., Teacher A18 stated, “Let students imitate movements and avoid confrontation”). This practice is inconsistent with the requirements of the “Efficiency Approach” or the combination of the two approaches preferred by the National School Martial Arts Alliance, representing a form of implicit resistance characterized by outward compliance and inward opposition. Theoretically, this phenomenon results from weak perceived behavioural control (safety concerns) and strong subjective normative pressure (administrative requirements) within the Theory of Planned Behavior. It also aligns with Fullan's notion of “symbolic implementation-type resistance”, reflecting that the absence of safety standard policies in SMAP leads teachers to orient their agency toward an “avoidance-oriented” approach (47). Mode 3: explicit opposition (Low Levels of Both Attitudinal Support and behavioural Participation)Teachers explicitly disagree with the SMAP philosophy and refuse to participate (e.g., Teacher A12 stated, “I do not agree with this philosophy and refuse to participate”). This reflects not only “negative attitudes” in the Theory of Planned Behavior but also corresponds to Fullan's concept of inertia-related resistance (47). Teachers have long been accustomed to traditional routine-based teaching; the combat-focused instruction required by SMAP demands the reconstruction of lesson plans and participation in training. The high cost of this reform exceeds their willingness to invest agency. Priestley et al. also pointed out that when reform requirements significantly narrow the projection space (e.g., requiring a complete shift from long-standing teaching habits), explicit opposition is likely to occur (38). Mode 4: structural constraint (High Attitudinal Support, Low behavioural Participation)Teachers recognize the value of SMAP but are unable to participate due to objective constraints (e.g., Teacher A19 acknowledged the program's value but could not teach it due to lack of training).Theoretically, this is a typical manifestation of “lack of perceived behavioural control” in the Theory of Planned Behavior. From Priestley et al.'s structural-agency perspective (38), although teachers possess value-driven agency (recognizing SMAP's value), they are constrained by structural barriers such as lack of training and insufficient hardware. As a result, their agency fails to translate into actual participation, highlighting deficiencies in supporting policies and insufficient structural support during the promotion of SMAP. From Fullan's (2016) perspective, this corresponds to what he describes as technical resistance. Bridwell-Mitchell also emphasized that training and hardware support are prerequisites for translating policy intentions into practice (48). Having clarified the central role of teacher participation in SMAP success, the next section shifts focus to the cognitive precursor of this participation—Teacher Evaluation—analyzing how teachers' assessments of difficulty, risk, value, and cost-benefit shape their engagement.

### 4.2 Analysis of teacher evaluation

Teacher Evaluation refers to the overall judgment made by teachers participating in martial arts teaching reform regarding the implementation of martial arts combat-oriented teaching, and it serves as a response to the question of how teachers view the integration of martial arts into school physical education curricula. By evaluating the difficulty, risk, value, and cost-benefit of SMAP, teachers form a basic assessment of whether martial arts are suitable for integration into the curriculum, thereby providing a cognitive foundation for determining what kind of response behaviours to adopt. This process not only aligns with the Theory of Planned Behavior's assertion that “behavioural attitudes are shaped by cognitive evaluation” (37), but also reflects the active cognitive construction of educational reform by teachers as agentic subjects (38). **In difficulty evaluation**, teachers focus on the adaptability of implementation conditions. A core finding is that teachers frequently mention the lack of professional martial arts training and insufficient hardware facilities as factors that increase the difficulty of SMAP implementation. When teachers perceive a mismatch between existing conditions and SMAP requirements, their acceptance of “integrating martial arts into the curriculum” decreases significantly. Theoretically, this represents a concrete manifestation of perceived behavioural control in the Theory of Planned Behavior—specifically, teachers' judgment of their ability to manage and execute the SMAP implementation process. It provides localized empirical support for understanding how condition adaptability shapes teachers' attitudes in physical education curriculum reform. **In risk evaluation**, teachers emphasize potential safety hazards and associated teaching responsibilities. Many express concern that SMAP's focus on combat skills may increase the likelihood of student injuries, thereby exposing teachers to greater professional liability. This reveals that teachers' concerns center on the trade-off between implementing the reform's pedagogical philosophy and fulfilling their duty of care. It further explains why some teachers adopt a wait-and-see attitude toward integrating martial arts into the curriculum. From a policy perspective, this underscores the urgency of teacher professional development and institutional support. Relevant authorities should establish specialized safety teaching standards for SMAP—such as simplifying combat movements and mandating protective equipment—while offering targeted training in safe martial arts instruction. These measures would support teachers' professional growth, reduce perceived risks, and lay a foundational basis for SMAP implementation in schools. **In value evaluation**, teachers assess the realization of educational value. While they acknowledge the core value of “emphasizing combat skills and nurturing character”, they remain skeptical about its practical translation. Specifically, teachers perceive character development as a long-term process, and believe that 1–2 semesters of martial arts instruction are insufficient to ensure measurable outcomes. Moreover, there is a cognitive gap in defining the core content of martial arts philosophy and identifying effective pathways to integrate it with combat teaching (5). Teachers also question whether martial arts instruction can be adapted to meet the needs of special student groups, such as those with disabilities or autism, thus raising concerns about inclusive educational effectiveness. Notably, Ciaccioni's intergenerational judo study has highlighted the lifelong educational value of martial arts (49), yet teachers' evaluations remain constrained to short-term instructional outcomes. They often fail to consider the broader, long-term contributions of martial arts to intergenerational connection, the cultivation of healthy exercise habits, and lifelong physical activity engagement. This limitation suggests that teachers' value evaluation should expand beyond immediate teaching effects to encompass the philosophical depth of martial arts, inclusivity across diverse student populations, and lifelong developmental impacts. To facilitate this shift, educational institutions should invite martial arts education experts to lead specialized teaching and research activities, support teachers in collaboratively developing “combat + philosophy” curricula tailored to different school levels, and collaborate with special education specialists and martial arts coaches to design adaptive martial arts programmes for special needs students—thereby deepening teachers' understanding of SMAP's full educational potential. **In cost-benefit evaluation**, teachers weigh the investment of time and effort against potential returns. Some teachers note that participation in SMAP requires additional time and energy for lesson preparation and training, yet there are no corresponding incentives—such as advantages in professional title evaluation or performance recognition. This helps explain why certain teachers, despite recognizing SMAP's value, remain unwilling to engage proactively: an imbalance between costs and benefits undermines their motivation. From a policy standpoint, this calls for schools and educational authorities to establish incentive mechanisms—for instance, incorporating SMAP teaching into performance evaluations or allocating dedicated teaching and research funding. At the same time, reducing teachers’ workload through collective lesson planning and shared teaching resources represents a critical institutional support strategy to enhance proactive teacher participation in SMAP.

### 4.3 Analysis of constraints on teachers' implementation

Teacher Implementation Constraints refer to the behavioural restrictions imposed by external conditions and relevant groups on teachers' participation in SMAP—guided by the concept of “emphasizing combat skills and nurturing character”. This construct responds directly to the study's research question—"what response behaviours teachers adopt toward SMAP"—by delineating how such constraints limit teachers' behavioural space, prevent the translation of behavioural intentions into actual participation, and constitute a key structural barrier in the promotion of SMAP. Teacher Implementation Constraints are categorized into two dimensions: implementation condition constraints and educational stakeholder constraints. Implementation condition constraints center on the combined effect of insufficient material conditions and lack of institutional support in limiting teacher participation. Teachers' competence in martial arts combat teaching is foundational for engaging with SMAP. A safe and standardized teaching system enhances the organization and delivery of instructional content. For example, Antunes’ framework, which classifies martial arts movements into “unarmed movements” and “weapon-based movements” based on shared characteristics, offers teachers a structured reference for curriculum organization (50). Comprehensive hardware facilities—such as protective gear and dedicated training venues—can also mitigate the risk of injuries during instruction (51). However, most teachers report a lack of these essential conditions, rendering them unable to implement SMAP even when they recognize its value. From policy and teacher professional development perspectives, progress requires intervention in two areas: First, strengthening teacher competence development—by integrating martial arts combat content into pre-service physical education teacher training curricula and establishing a SMAP-specific in-service training system; second, enhancing institutional support—through the allocation of special funds to upgrade hardware (e.g., protective equipment, dedicated venues) and the standardization of teaching content frameworks to provide teachers with clear instructional guidance. Institutional constraints further compound these participation barriers. Currently, there is a lack of established in-service training programmes and teaching seminars to support SMAP implementation, making it difficult for teachers to acquire the subject-specific content knowledge and practical skills required for martial arts instruction (52). At the same time, the absence of regional martial arts competitions or leagues focused on combat skills for ordinary students (53) deprives SMAP of platforms for demonstrating outcomes. Without visible results or performance channels, school leaders are less likely to perceive the program's value, which in turn reduces their willingness to allocate resources and support. This indicates that, at the policy level, it is necessary to establish a regular system of teaching and research training for SMAP, and simultaneously promote the development of a regional student martial arts competition system. By creating a platform for the transformation and visibility of project outcomes, the perceived value of SMAP can be enhanced among school administrators, thereby increasing institutional support and enabling more sustainable program implementation.

In terms of constraints from educational stakeholder groups, the attitudes of school leaders, parents, and students directly affect teachers' actual participation behaviours in SMAP, and “ensuring student safety” is a shared view among the three groups (e.g., as mentioned by Teacher A2, “The implementation of martial arts combat teaching must ensure student safety; otherwise, no one would agree”). Specifically: School leaders focus on whether SMAP can highlight the school's distinctive martial arts education features and additional value, but they worry about teachers' martial arts professional qualifications and the potential for combat sports to trigger aggressive behaviours among students (54); moreover, under China's hierarchical school management system, their attitudes directly determine whether teachers can obtain the authority to implement SMAP. Parents care about whether SMAP can bring developmental value to their children, such as expending excess energy to improve learning focus, learning self-defense skills to deal with emergencies, and cultivating positive character traits (55); students, on the other hand, are more concerned about whether the martial arts taught by teachers possess combat applicability, as they are reluctant to engage in simple and tedious martial arts routine content. In response to this, policy and institutional support need to take targeted measures: incorporate SMAP into the school's characteristic curriculum planning to gain school leaders' support, organize parent lectures to explain SMAP's safety standards and educational value to alleviate parents' concerns, and optimize the design of teaching content (integrating practical combat elements) to align with students' interests, thereby creating a favorable external environment for teachers' participation in SMAP.

## 5 Conclusions and future directions

### 5.1 Conclusions

The core theoretical contribution of this study lies in clarifying that teacher participation is the decisive factor for the successful implementation of the School Martial Arts Program (SMAP). Specifically, when confronted with the SMAP philosophy of “emphasizing combat skills and nurturing character”, teachers do not directly accept or reject it outright; instead, they form differentiated participation modes through the dynamic interaction of “four-dimensional evaluation (difficulty-risk-value-cost-benefit)” and “dual constraints (educational stakeholders-implementation conditions)”. Among these modes, teacher resistance—comprising implicit resistance, explicit opposition, and structural constraint—acts as a barrier that hinders the realization of SMAP's educational value. Secondary insights of this study include: first, teachers' evaluation of SMAP exhibits a “short-term effect orientation”, meaning they tend to overlook the lifelong value of martial arts; second, among implementation constraints, “insufficient teaching competence and hardware facilities” and “lack of specialized training” emerge as the most prominent bottlenecks.

Based on the aforementioned findings, the practical implications for the promotion of SMAP are as follows: At the policy level, it is necessary to establish a SMAP-specific incentive mechanism (e.g., incorporating martial arts teaching into teacher performance evaluation, allocating special funds for teaching and research), formulate safety operation standards for combat teaching (e.g., simplifying confrontational movements, equipping protective gear), and improve the regional student martial arts competition system (e.g., combat-oriented competitions). The demonstration of program outcomes can enhance school leaders' willingness to support SMAP. At the teacher education level, pre-service physical education teacher training should integrate martial arts combat teaching content; for in-service teachers, it is essential to establish a SMAP-specific training and teaching research system (e.g., collective lesson preparation, sharing of “combat + philosophy” lesson plans) to address the shortage of teachers' martial arts teaching competence. At the curriculum reform level, there is a need to develop integrated courses of “combat skills + traditional culture” by school stage, and to collaborate with special education experts and martial arts coaches to design adaptive courses for students with disabilities (e.g., VR-assisted martial arts teaching), so as to respond to the demand for educational inclusiveness.

### 5.2 Future directions

First, focus on diversity and inclusivity: Explore the collaborative teaching mechanism of martial arts between martial arts teachers and adapted physical education teachers (e.g., adaptive movement design, hierarchical protection plans); analyze how SMAP connects the primary to senior high school stages, construct an intergenerational martial arts education pathway through the linkage of “school-family-community” (e.g., parent-child martial arts courses), and examine the sustained value of martial arts across cross-age groups. Second, improve research methods: Conduct quantitative research (e.g., a nationwide questionnaire survey targeting physical education teachers in primary and secondary schools) to investigate the distribution characteristics of the four participation modes and their influencing factors (e.g., the impact of teaching seniority and martial arts background on participation attitudes); alternatively, carry out cross-cultural comparisons (e.g., comparing teacher participation differences in China, South Korea, and Japan regarding school taekwondo and karate programmes) to validate the generalizability of the grounded theory model proposed in this study. Third, expand research dimensions: Introduce contextual variables such as policy implementation levels (national, local, school) and school types (public, private) to further enrich the framework of factors influencing teacher participation.

### 5.3 Research limitations

Singularity of Research Methods: The study lacks quantitative research support, making it difficult to quantify the relative impact of different constraining factors—such as insufficient hardware facilities and parents' attitudes—on teachers' participation in SMAP. Incomplete Variable Coverage: Furthermore, “local differences” in policy implementation—such as variations in the level of support for SMAP from regional education authorities and discrepancies in the details of policy execution—have not been fully incorporated into the analysis, resulting in a less comprehensive understanding of the full range of factors constraining teacher participation.

## Data Availability

The original contributions presented in the study are included in the article/Supplementary Material, further inquiries can be directed to the corresponding author.
